# Clinical characteristics with long-term follow-up of four Okinawan families with moderate hearing loss caused by an *OTOG* variant

**DOI:** 10.1038/s41439-019-0068-4

**Published:** 2019-08-13

**Authors:** Akira Ganaha, Tadashi Kaname, Kumiko Yanagi, Tetsuya Tono, Teruyuki Higa, Mikio Suzuki

**Affiliations:** 1 Department of Otorhinolaryngology-Head and Neck Surgery, University of Miyazaki, Miyazaki, Japan; 2 Department of Human Genetics, National Center for Child Health and Development, Tokyo, Japan; 3 Department of Otorhinolaryngology-Head and Neck Surgery, University of the Ryukyus, Okinawa, Japan

**Keywords:** Medical genetics, Diseases

## Abstract

We describe the clinical features of four Japanese families with moderate sensorineural hearing loss due to the *OTOG* gene variant. We analyzed 98 hearing loss-related genes in patients with hearing loss originally from the Okinawa Islands using next-generation sequencing. We identified a homozygous variant of the gene encoding otogelin NM_001277269(OTOG): c.330C>G, p.Tyr110* in four families. All patients had moderate hearing loss with a slightly downsloping audiogram, including low frequency hearing loss without equilibrium dysfunction. Progressive hearing loss was not observed over the long-term in any patient. Among the three patients who underwent newborn hearing screening, two patients passed the test. *OTOG*-associated hearing loss was considered to progress early after birth, leading to moderate hearing loss and the later stable phase of hearing loss. Therefore, there are patients whose hearing loss cannot be detected by NHS, making genetic diagnosis of *OTOG* variants highly useful for complementing NHS in the clinical setting. Based on the allele frequency results, hearing loss caused by the p.Tyr110* variant in *OTOG* might be more common than we identified. The p.Tyr110* variant was reported in South Korea, suggesting that this variant is a common cause of moderate hearing loss in Japanese and Korean populations.

## Hearing loss: Newborn hearing tests can miss inherited OTOG deficiency

People born with mutations in a gene linked to moderate hearing loss don’t always show problems during newborn hearing screening and may benefit from genetic screening. Testing for this gene, *OTOG*, which encodes a protein expressed in sensory cells of the inner ear, could thus be an important complement to newborn hearing screening for individuals with a family history of hearing loss who may benefit from early medical intervention. A Japan-based team led by Akira Ganaha from the University of Miyazaki studied seven patients with hearing loss from four unrelated families living on the Japanese archipelago of Okinawa. Each had two copies of a recessive mutation in *OTOG*, a variant previously documented only in South Korea. Of the three who underwent newborn hearing tests, only one was diagnosed with hearing deficiency at birth.

## Introduction

To date, over 100 causative genes have been identified for nonsyndromic deafness, of which 70% follow a recessive inheritance pattern^1,2^. A large majority of recessive variants are associated with congenital, nonprogressive, and severe-to-profound hearing loss^3^. Compared with severe-to-profound hearing loss, mild-to-moderate hearing loss in children has been less well characterized. *OTOG* (DFNB18B) is a causative gene of mild-to-moderate nonsyndromic hearing loss^4,5^. *OTOG* encodes otogelin, which is a noncollagenous protein specific to the inner ear^6^. Hearing loss in *OTOG* knockout mice is bilateral and highly progressive immediately after birth, ranging from mild-to-profound^7^. Five causative variants in *OTOG* have been reported in four families to date^4,5,8^. Although only four families have been reported to date with *OTOG* mutations, the onset and long-term course of hearing loss and complications of disequilibrium have not been clarified.

We analyzed 98 genes related to hearing loss using next-generation sequencing (NGS) in patients originally from the Okinawa Islands. NGS revealed the presence of a homozygous variant in exon 4 of *OTOG*, c.330C>G, p.Tyr110*, in four families. In this study, we investigated the onset and long-term clinical features of hearing loss patients with the *OTOG* p.Tyr110* variant. Based on the allele frequency in the 1000 Genomes Project and in this study, hearing loss caused by the *OTOG* gene might be more common than reported previously.

## Materials and methods

### Subjects

**Fig. 1:**
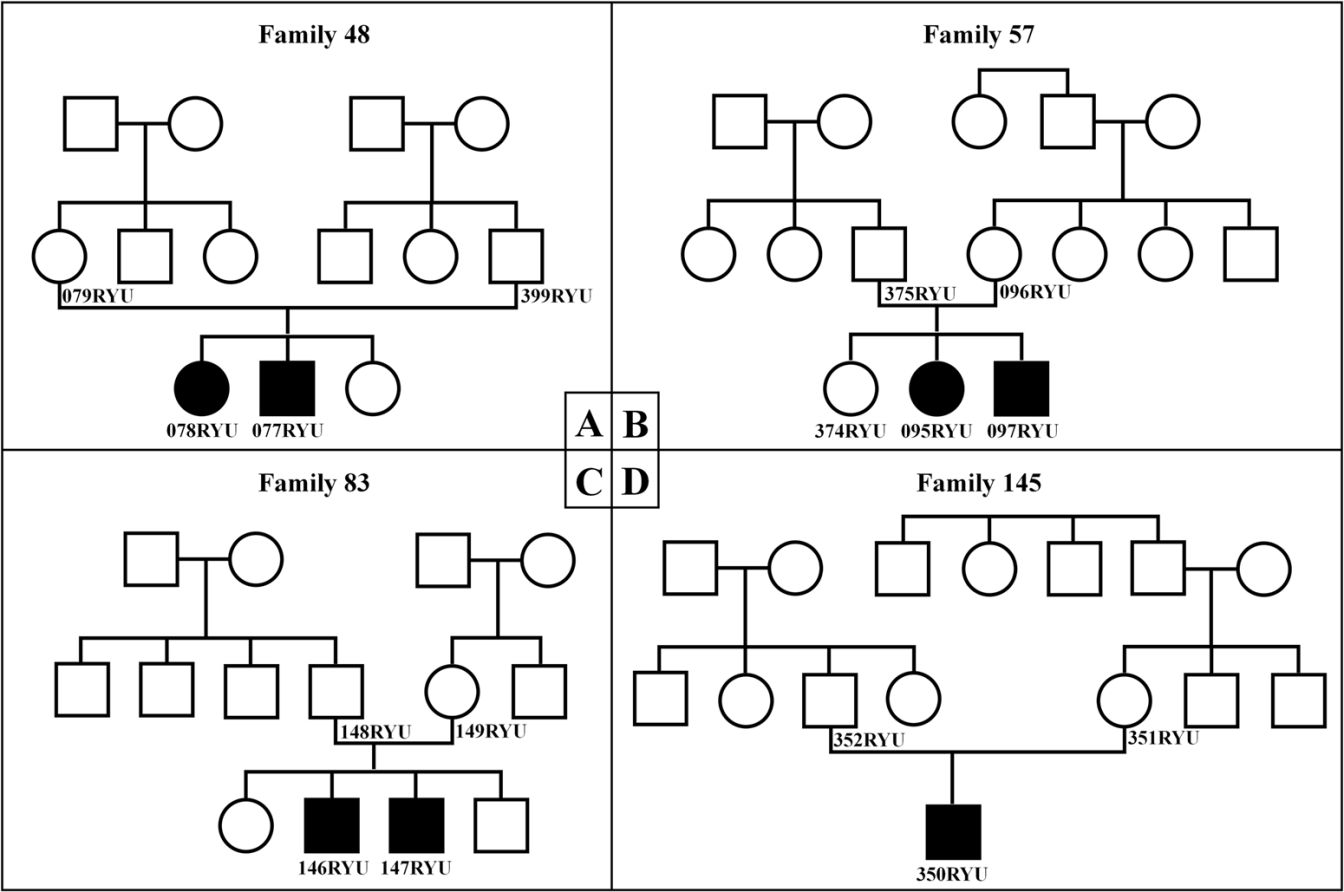
Pedigree of the seven reported patients. Pedigrees of (**a**) patient 078RYU (III-1) and 077RYU (III-2), (**b**) patient 095RYU (III-2) and 097RYU (III-3), (**c**) patient 146RYU (III-2) and 147RYU (III-3), and (**d**) patient 350RYU (III-1). Hearing loss occurred in an autosomal recessive inheritance pattern

Seven affected patients from four unrelated families were investigated (Fig. 1). Clinical history was taken from all patients, and physical examinations were performed, including otoscopy, hearing tests, and computed tomography (CT) of the temporal bones. Depending on each patient’s ability, hearing level was determined using auditory steady-state response (ASSR), auditory brainstem response (ABR), conditioned orientated response, or pure tone audiogram (PTA). Hearing level was defined as the average hearing threshold at 0.5, 1.0, 2.0, and 4.0 kHz. Hearing was described as follows: normal, < 20 dB; mild impairment, 21–40 dB; moderate impairment, 41–70 dB; severe impairment, 71–90 dB; and profound impairment, > 91 dB.

Prior to enrollment, all subjects provided written informed consent. Our research protocol was approved by the Ethics Review Boards of the University of Miyazaki and University of the Ryukyus.

### *OTOG* genotyping

#### NGS

Targeted resequencing for hearing loss (family 48) or whole-exome sequencing (family 57, 83, 145) was performed as described previously^9,10^. Briefly, genomic DNA was extracted from whole-blood cells using the QIAamp DNA Blood Mini Kit (QIAGEN, Hilden, Germany). For targeted resequencing, a customized HaloPlex Target Enrichment Panel (Agilent Technologies, Santa Clara, CA, USA) was used to enrich each DNA sample from the families for 98 candidate genes for hearing loss, including *OTOG*. Each enriched DNA samples were sequenced with the MiSeq System (Illumina, San Diego, CA, USA). Variants from sequencing data were called using the SureCall pipeline (Agilent Technologies). For whole-exome sequencing analysis, the SureSelect Human All Exon V6 Kit (Agilent Technology, Santa Clara, CA, USA) was used for capture, and the HiSeq2500 (Illumina, San Diego, CA, USA) was used for sequencing. Reads were aligned to CRC37 using Burrows-Wheeler Aligner. Variants were called using the GATK Unified Genotyper and ANNOVA (http://annover.openbioinfomatics.org/en/latest/).

#### Sanger sequencing

To confirm the coding sequence and variant, Sanger sequencing of the *OTOG* gene was performed in all subjects. PCR was performed as follows: initial denaturation at 94 °C for 5 min; 35 cycles of 94 °C for 40 s, 64 °C for 40 s, and 72 °C for 1 min; and a final extension at 72 °C for 5 min. PCR was performed on a programmable thermal cycler (Verti 96-Well Thermal Cycler; Applied Biosystems, Foster City, CA, USA). PCR products were purified using the Wizard SV Gel and PCR Clean-Up System (Promega, Madison, WI, USA) and sequenced directly using an ABI PRISM 3130xl Genetic Analyzer (Applied Biosystems). The sequences were aligned and compared using the BLAST program with known human genome sequences available in the GenBank database.

#### PCR–restriction fragment length polymorphism (RFLP) analysis of *OTOG*

We surveyed the substitutions in exon 4 of *OTOG* (c.330C>G, p.Tyr110*) in 100 healthy unrelated Okinawan individuals used as controls. The genotypes of the p.Tyr110* variants were detected by digestion of the PCR product with the restriction enzyme *Bsa*XI (New England BioLabs, Ipswich, MA, USA).

## Results

**Fig. 2:**
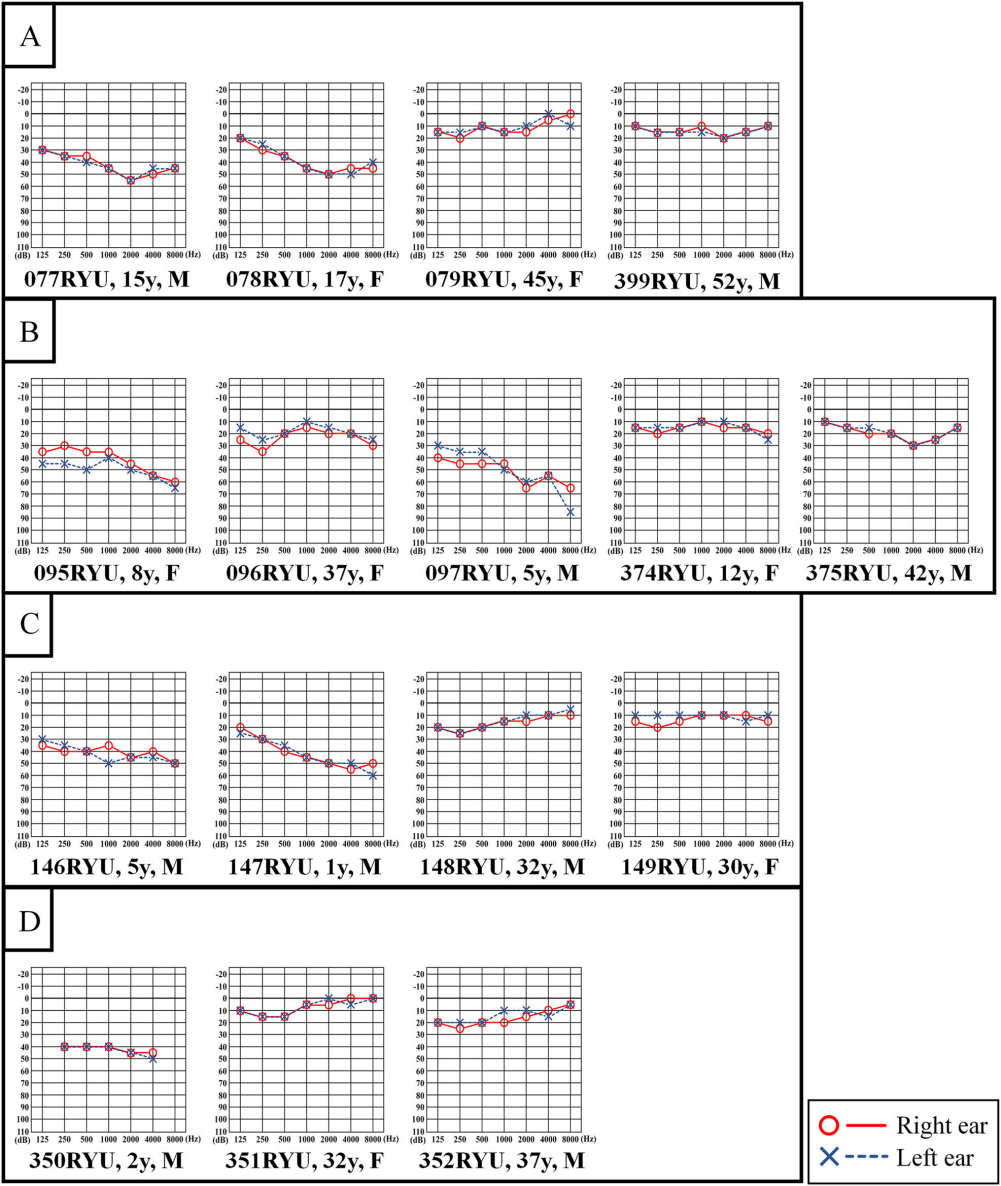
Pure tone audiometry of the patients and family members. **a** Audiograms in family 48, (**b**) family 57, (**c**) family 83, and (**d**) family 145 indicate moderate hearing loss in the patients. Hearing thresholds for the right ear are represented by red circles, and the thresholds for the left ear are represented by the blue Xs

NGS identified a homozygous variant in exon 4 of the *OTOG* gene, c.330C>G, p.Tyr110*, in seven patients with moderate sensorineural hearing loss (Figs. 1, 2).

Table 1 shows the clinical features and the results of examinations in all patients. Newborn hearing screening (NHS) was performed in three patients; two of these patients passed the NHS. PTA showed moderate sensorineural hearing loss with a slightly downsloping pattern involving low frequency loss in all patients (Fig. 2). Distortion product otoacoustic emissions (DPOAE) showed no response in all patients (Table 1) at their first visit to our hospital. ABR or ASSR was performed in six of the seven patients, which showed consistent results with the PTA (Table 1). No patient exhibited vertigo or abnormal findings in the middle or inner ear on temporal bone high-resolution CT (Table 1). Five of the seven patients had been using bilateral hearing aids. None of the patients showed progression of their hearing loss during the follow-up period (100 ± 68 months). In particular, two patients (077RYU and 078RYU in Table 1) did not show progression of hearing loss for more than 10 years. An articulation test was performed in six of the seven patients. At their first visit to our hospital, all patients were observed to be developing normally in areas unrelated to speech, language, and hearing. All six patients with the *OTOG* variant demonstrated fewer articulation errors at the age of 4 or 5 years. However, four patients aged 10 years and over demonstrated an improvement in articulation skills with age regardless of the presence or absence of a hearing aid.

**Table 1:** Summary of the clinical findings of the seven patients

Family	Patient	Age at first visit (years)	Sex	NHS		First PTA		Latest PTA		DPOAE		ABR		ASSR								Follow-up period (months)	Vertigo	CT findings	HA
				Rt.	Lt.	Rt. (dB)	Lt. (dB)	Rt. (dB)	Lt. (dB)	Rt.	Lt.	Rt. (dB)	Lt. (dB)	Rt. (dB)				Lt. (dB)							
														500	1 k	2 k	4 k	500	1 k	2 k	4 k				
48	077RYU	7	M	NA		46	45	47	45	Negative	Negative	NA		NA				NA				155	No	No abnormality	Yes
	078RYU	3	F	NA		47	45	43	43	Negative	Negative	50	60	NA				NA				232	No	No abnormality	Yes
57	095RYU	5	F	NA		42	42	38	45	Negative	Negative	50	50	40	40	40	50	40	40	50	50	76	No	No abnormality	No
	097RYU	2	M	NA		47	51	51	48	NA	NA	60	60	60	50	50	60	60	50	50	60	70	No	No abnormality	Yes
83	146RYU	4	M	Pass	Pass	40	40	40	45	Negative	Negative	40	50	50	50	50	40	50	50	50	50	56	No	No abnormality	No
	147RYU	0	M	Pass	Refer	41	45	41	41	Negative	Negative	40	50	40	30	40	40	40	40	50	50	61	No	No abnormality	Yes
145	350RYU	0	M	Refer	Refer	41	41	41	41	Negative	Negative	50	60	40	50	50	50	50	60	50	60	47	No	No abnormality	Yes

*NHS* newborn hearing screening, *PTA* pure tone audiometry, *DPOAE* distortion product otoacoustic emissions, *ABR* auditory brainstem response, *ASSR* auditory steady-state response, *HA* hearing aid, *Rt*. right, *Lt.* left, *NA* not assessed

**Fig. 3:**
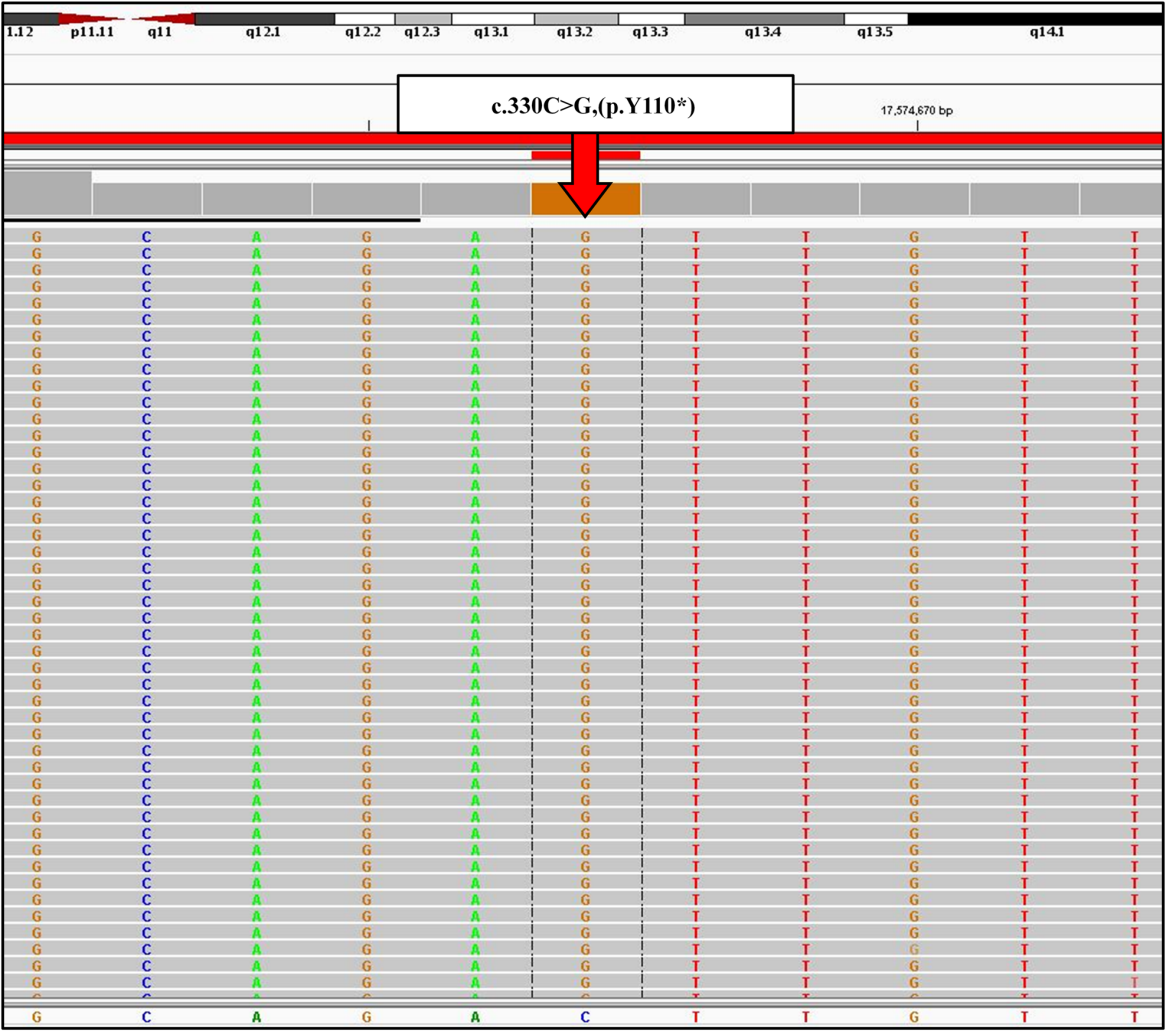
Integrative Genomics Viewer images of NGS analysis of the *OTOG* gene. Integrative Genomics Viewer images of NGS analysis of the *OTOG* gene (forward sequence) for 077RYU (family 48). The arrow indicates the variant nucleotide. The reference sequence is presented at the bottom of Fig. 3

**Fig. 4:**
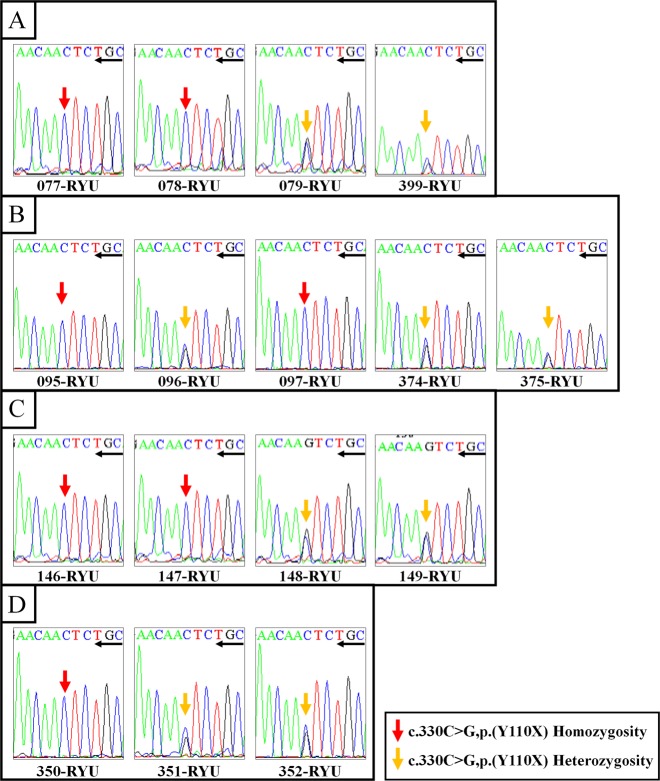
Examples of Sanger sequence analysis of the *OTOG* gene. *OTOG* gene sequencing profiles of the patients. Sanger sequencing diagram (reverse sequence) for (**a**) family 48, (**b**) family 57, (**c**) family 83, and (**d**) family 145. Red arrows indicate the homozygous variant of p.Tyr110X, yellow arrows indicate the heterozygous variant

Figure 3 shows the results of NGS of the *OTOG* gene variant, c.330C>G, p.Tyr110*, on the Integrative Genome Viewer. The presence of the homozygous variant of p.Tyr110* in patients and the heterozygous variant in their parents was confirmed by direct sequencing analysis (Fig. 4). Allele frequency analysis of 100 control individuals with normal hearing using PCR-RFLP revealed one heterozygous variant of p.Tyr110*, but no homozygous variants (data not shown).

## Discussion

*OTOG* (DFNB18B) is a causative gene of nonsyndromic mild-to-moderate hearing loss that follows an autosomal recessive inheritance pattern. Five causative variants in *OTOG* have been reported in four families to date^4,5,8^ (Table 2). *OTOG* encodes otogelin protein and is expressed in the sensory epithelium of the inner ear, including the tectorial membrane, otoconial membranes in the utricle and saccule, and cupula that covers the crista ampullaris of the semicircular canal in the vestibular organ^6^. In the cochlea, *OTOG* appears to be involved in organizing the fibrillar network of the tectorial membrane and likely plays a role in determining the resistance of this membrane to sound stimulation^7^. Hearing loss in *OTOG* knockout mice is bilateral and highly progressive immediately after birth, ranging from mild-to-profound^7^. However, it has not been clarified whether *OTOG*-associated hearing loss in humans is congenital or progresses early after birth. In this study, NHS was performed using automated auditory brainstem responses in three patients, and two patients passed the NHS. In both patients, moderate hearing loss of more than 40 dB was confirmed by hearing tests including PTA, DPOAE, ABR, and ASSR at the first visit of our hospital. These results indicate that hearing loss due to *OTOG* variants is not congenital, but that hearing loss immediately after birth may be genetically determined. There is a risk that patients with *OTOG* variants may pass the NHS and miss the opportunity to receive early medical intervention for hearing loss. Therefore, accurate diagnosis of such genetic variants is clinically useful to complement NHS and prevent overlooking hearing loss that develops immediately after birth.

**Table 2:** Clinical findings of previously described and our families

	Schraders et al.^4^		Danial-Farran N^8^	Yu et al.^5^ (2018)	Our families			
Family	Dutch family	Spanish family	Arab family	Korean family	Japanese family			
					Family 48	Family 57	Family 83	Family 145
Variant	c.5508delC, p.(Ala1838Profs*31)	c.6347C>T,p.(Pro2116Leu) / c.6559C>T,p.(Arg2187*)	c.7453C>T,p.(Arg2485*)	c.330C>G,p.(Tyr110*)	c.330C>G, p.(Tyr110*)			
	Homozygous	Compound heterozygous	Homozygous	Homozygous	Homozygous			
Severity of Hearing loss	Mild-to-moderate	Mild-to-moderate	NA	Mild	Moderate			
Shape of audiogram	Flat to shallow U-shaped	Slightly downsloping	NA	Slightly downsloping	Slightly downsloping			
Vestibular dysfunction^a^	+	+	NA	−	−	NA	NA	NA
Symptom of Vertigo^b^	Delayed motor development	−	NA	−	−			

^a^The results of equilibrium function test using caloric test and/or rotary chair test

^b^Clinical episode of vertigo or suspected vestibular dysfunction

Hearing loss due to *OTOG* variants shows a slightly downsloping audiogram or a U-shaped to flat audiogram, and the pattern is similar to that of hearing loss due to variants in *TECTA*, which is also expressed in the tectorial membrane^4^. In the present report, a slightly downsloping shaped audiogram was detected in all patients, and an increase in the low frequency threshold was especially characteristic. In addition, based on the observation of the clinical course of the same patients’ hearing over 10 years, it was clinically determined that hearing loss due to p.Tyr110* is not progressive over time. Thus, we determined that hearing loss due to p.Tyr110* is characterized by onset immediately after birth, leading to moderate hearing loss with a slightly downsloping shaped audiogram with nonprogressive features.

Equilibrium dysfunction and associated motor developmental delay have been reported in patients with *OTOG* variants^4^. A previous study reported that vestibular dysfunction was present in *OTOG* knockout mice^7^. None of our seven patients had episodes of vertigo or motor developmental delay (Table 2). A caloric test could be performed in only one patient (077RYU); however, no obvious canal paresis was observed. A previous report with the same genetic variant as our report indicated that there was no equilibrium dysfunction in the patients^5^. The possibility cannot be excluded that the differences in phenotype between the previous report^4^ and the present report are associated with different variants. However, the genotype–phenotype correlation is not clear, as only eight families, including our four families, with hearing loss caused by *OTOG* variants have been reported to date.

Among the hereditary hearing loss, the c.235delC in *GJB2* gene was identified as the most frequent pathogenic variant, followed by p.H723R in *SLC26A4* in Japanese population^11^. The allele frequency of c.235delC in *GJB2* is 1% and that of the p.H723R in *SLC26A4* is 0.5% in the 1000 Genomes Project (1000G). The p.Tyr110* variant was reported in only the Japanese population in the 1000G with an allele frequency of 0.5%. In this study, the allele frequency of the p.Tyr110* in Okinawan control individuals was identified in 0.5% (1/200), with the same allele frequency in 1000G and that of p.H723R in *SLC26A4*. Which means that one out of 40,000 may have mild-to-moderate hearing loss due to the p.Tyr110*. Based on these results, hearing loss caused by p.Tyr110* in *OTOG* might be more common than we identified. However, only eight families, including our four families, with hearing loss caused by *OTOG* variants have been reported to date, and this is the first report of hearing loss caused by this variant in the Japanese population. There is a possibility of underestimation of the prevalence of hearing loss caused by the *OTOG* gene in that the patients with the variant might be excluded from the genomic research because of mild phenotype^12^.

The Okinawa Islands are the southwestern-most islands of the Japanese archipelago. Previous studies suggested that there were substantial ancestral differences between the Okinawa Islands and the main islands of Japan^13^. The common variant p.Tyr110* identified in all four families in this report is considered to be the result of a founder effect by a common ancestor. The same variant was reported from Korea in 2018^5^, which also suggests that the variant has common ancestor in Korea^5^. Altogether, it is possible that p.Tyr110* may be a common cause of moderate hearing loss in Japanese and Korean^5^ populations.

In conclusion, we identified the variant c.330C>G, p.Tyr110* in *OTOG* in seven patients of four families with moderate hearing loss in the Japanese population. Hearing loss was considered to occur immediately after birth, showing a moderate and slightly downsloping audiogram, and was nonprogressive thereafter. None of the families had symptoms of vestibular disorders. NM_001277269(OTOG): c.330C>G, p.Tyr110* was considered to be a highly frequent causative variant of mild-to-moderate hearing loss in Japanese and Korean^5^ populations. The present results with a long-term follow-up will improve the genetic counseling of patients with mutations in *OTOG*.
